# Solochrome cyanine: A histological stain for cobalt-chromium wear particles in metal-on-metal periprosthetic tissues

**DOI:** 10.1007/s10856-019-6304-0

**Published:** 2019-09-06

**Authors:** I. Papadimitriou-Olivgeri, J. M. Brown, A. F. R. Kilpatrick, H. S. Gill, N. A. Athanasou

**Affiliations:** 1 Department of Histopathology, NDORMS, University of Oxford, Nuffield Orthopaedic Centre, Oxford, OX3 7HE UK; 2 Chemistry Research Laboratory, Mansfield Road, Oxford, OX1 3TA UK; 3 Department of Mechanical Engineering, University of Bath, Bath, BA2 7AY UK

## Abstract

Metal-on-metal (MoM) hip arthroplasties produce abundant implant-derived wear debris composed mainly of cobalt (Co) and chromium (Cr). Cobalt-chromium (Co-Cr) wear particles are difficult to identify histologically and need to be distinguished from other wear particle types and endogenous components (e.g., haemosiderin, fibrin) which may be present in MoM periprosthetic tissues. In this study we sought to determine whether histological stains that have an affinity for metals are useful in identifying Co-Cr wear debris in MoM periprosthetic tissues. Histological sections of periprosthetic tissue from 30 failed MoM hip arthroplasties were stained with haematoxylin-eosin (HE), Solochrome Cyanine (SC), Solochrome Azurine (SA) and Perls’ Prussian Blue (PB). Sections of periprosthetic tissue from 10 cases of non-MoM arthroplasties using other implant biomaterials, including titanium, ceramic, polymethylmethacrylate (PMMA) and ultra-high molecular weight polyethylene (UHMWP) were similarly analysed. Sections of 10 cases of haemosiderin-containing knee tenosynovial giant cell tumour (TSGCT) were also stained with HE, SC, SA and PB. In MoM periprosthetic tissues, SC stained metal debris in phagocytic macrophages and in the superficial necrotic zone which exhibited little or no trichrome staining for fibrin. In non-MoM periprosthetic tissues, UHMWP, PMMA, ceramic and titanium particles were not stained by SC. Prussian Blue, but not SC or SA, stained haemosiderin deposits in MoM periprosthetic tissues and TSGT. Our findings show that SC staining (most likely Cr-associated) is useful in distinguishing Co-Cr wear particles from other metal/non-metal wear particles types in histological preparations of periprosthetic tissue and that SC reliably distinguishes haemosiderin from Co-Cr wear debris.

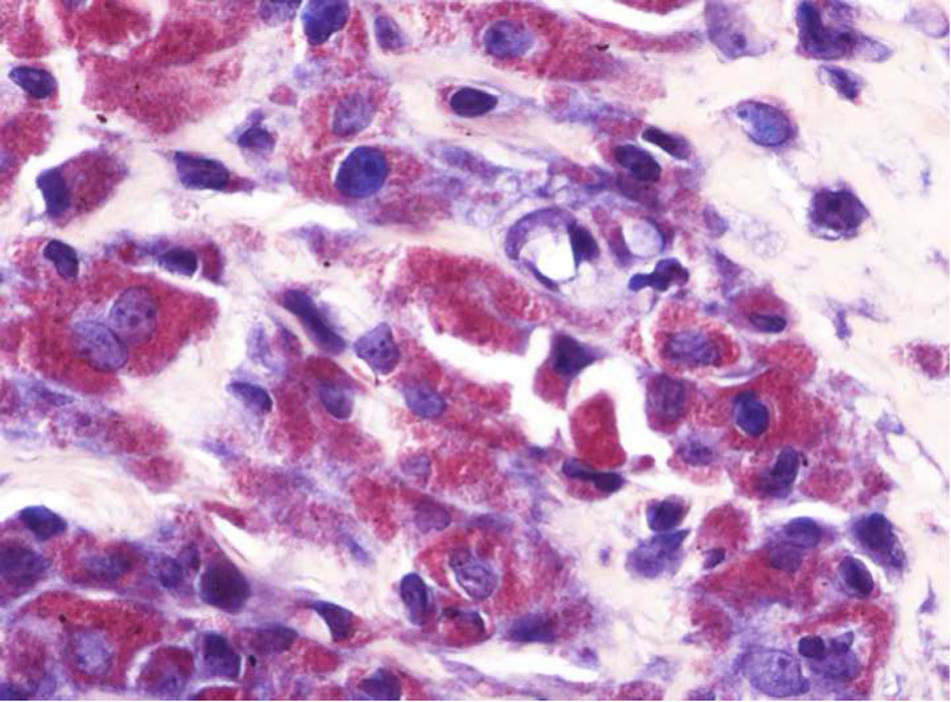

## Introduction

Metal-on-metal (MoM) implant components are composed of approximately 62% cobalt, 27–30% chromium, 5–7% molybdenum by weight with small amounts of nickel, iron, silicon, carbon and manganese [1–4]. Resurfacing and total MoM hip arthroplasties produce abundant cobalt-chromium (Co-Cr)-containing metal wear debris in both particulate and soluble forms. In periprosthetic tissue, particulate Co-Cr wear debris is phagocytosed by macrophages and macrophage polykaryons (giant cells) as part of the innate immune response to foreign material [3, 5–8]. Co-Cr particles and Co and Cr ions are cytotoxic and can elicit an adaptive cell-mediated immune response; as a consequence, there is marked necrosis and inflammation in periprosthetic tissues and MoM arthroplasties have been associated with early implant failure and pseudotumour formation [5–10].

Although MoM articulations generate less volumetric wear than non-MoM articulations, the number of wear particles produced is significantly greater as the particles are mainly nanometer in size [1–3]. It has been estimated that MoM articulations generate 6.7 × 10^12^ to 2.5 × 10^14^ particles every year [11]. Co-Cr particles are too tiny to be visible under the light microscope but small aggregates of these particles or shards of the metal implant can be identified histologically in MoM periprosthetic tissues [12, 13]. In routinely stained (haematoxylin-eosin) histological sections, Co-Cr particles within the cytoplasm of phagocytic cells may appear translucent/pink, slate blue/grey, green/yellow or brown/black in colour; this variable morphology may reflect differences in particle size/aggregation and/or corrosion and can make it difficult to identify Co-Cr particles with certainty in histological preparations [12–14]. Non-MoM total hip arthroplasties that employ a modular Co-Cr femoral stem also generate metal wear debris and uncommonly this can cause similar (MoM-associated) pathology and complications [15, 16].

Accurate identification of implant-derived wear particles in periprosthetic tissues is important in the histological assessment of implant failure [12–14]. Co-Cr particles need to be distinguished from wear particles derived from other metal or polymer implant components as well as endogenous metal/ non-metal tissue products such as haemosiderin and fibrin. There are a number of histological stains which have a particular affinity for metals including Perls’ Prussian Blue (PB), which is commonly used to identify iron (III) haemosiderin, and Solochrome Azurine (SA), which is used to identify aluminium [17]. Solochrome Cyanine (SC) is an organic reagent which has been used to identify chromium (III) by spectrophotometry [18]. Chrome azurol S (CAS), which is structurally related to SC, has been reported to identify chromium in histological sections in an experimental in-vivo model [19]. In this study, we have examined the diagnostic utility of SC and other metal-reacting histological stains to identify Co-Cr wear debris in MoM periprosthetic tissues. We have determined whether these stains react with other wear particle types in non-MoM periprosthetic tissues and have assessed whether endogenous products, notably fibrin and haemosiderin, are stained by these reagents.

## Materials and methods

Representative sections of the hip capsule, femoral and acetabular membrane were obtained from 30 cases of failed (aseptic) MoM hip resurfacing /total hip arthroplasty. Specimens from 10 cases of failed non-MoM hip arthroplasties employing ultra-high molecular weight polyethylene (UHMWP), polymethylmethacrylate (PMMA), ceramic and titanium components were similarly examined. Representative sections from 10 cases of haemosiderin-containing diffuse tenosynovial giant cell tumour (TSGCT) were also examined.

All specimens were fixed in 10% neutral buffered formalin, routinely processed and paraffin-embedded sections mounted on glass slides. The sections were cut at 4 µm. All sections were stained with Haematoxylin-eosin (HE), Perls’ Prussian blue (PB), Solochrome Cyanine (SC) and Solochrome Azurine (SA). Selected sections containing abundant exudate on the surface of MoM periprosthetic tissue were stained with Martius Scarlet Blue and Masson Goldners Trichrome, both trichrome stains for fibrin [17].

Staining methods employed for HE, SC, SA, and PB were as described by Bancroft and Stevens [17]. The method of SC staining is described below in detail but it is essentially unchanged from that described originally [20]. The SC staining solution was made up of 0.2 g of Solochrome Cyanine R and 4 ml of 10% diluted ammonium iron (III) sulphate dodecahydrate dissolved in 100 ml distilled water. The pH of the staining solution was adjusted to 1 by the addition of 0.5 ml sulphuric acid. The mixture was shaken vigorously and used immediately. The sections were stained with the staining solution for 20 min at room temperature. After rinsing in tap water, the sections were differentiated in 10 percent ammonium iron until nuclei were scarcely visible. Solochrome Cyanine variably stains cells and tissues blue/violet [17, 20, 21]. After washing in running tap water, the sections were counterstained with 1 percent neutral red for 5 min and then blot dried; the stained sections before being dehydrated through two changes of absolute alcohol and xylene before being coverslipped.

## Results

### Histological staining of MoM periprosthetic tissues

**Fig. 1:**
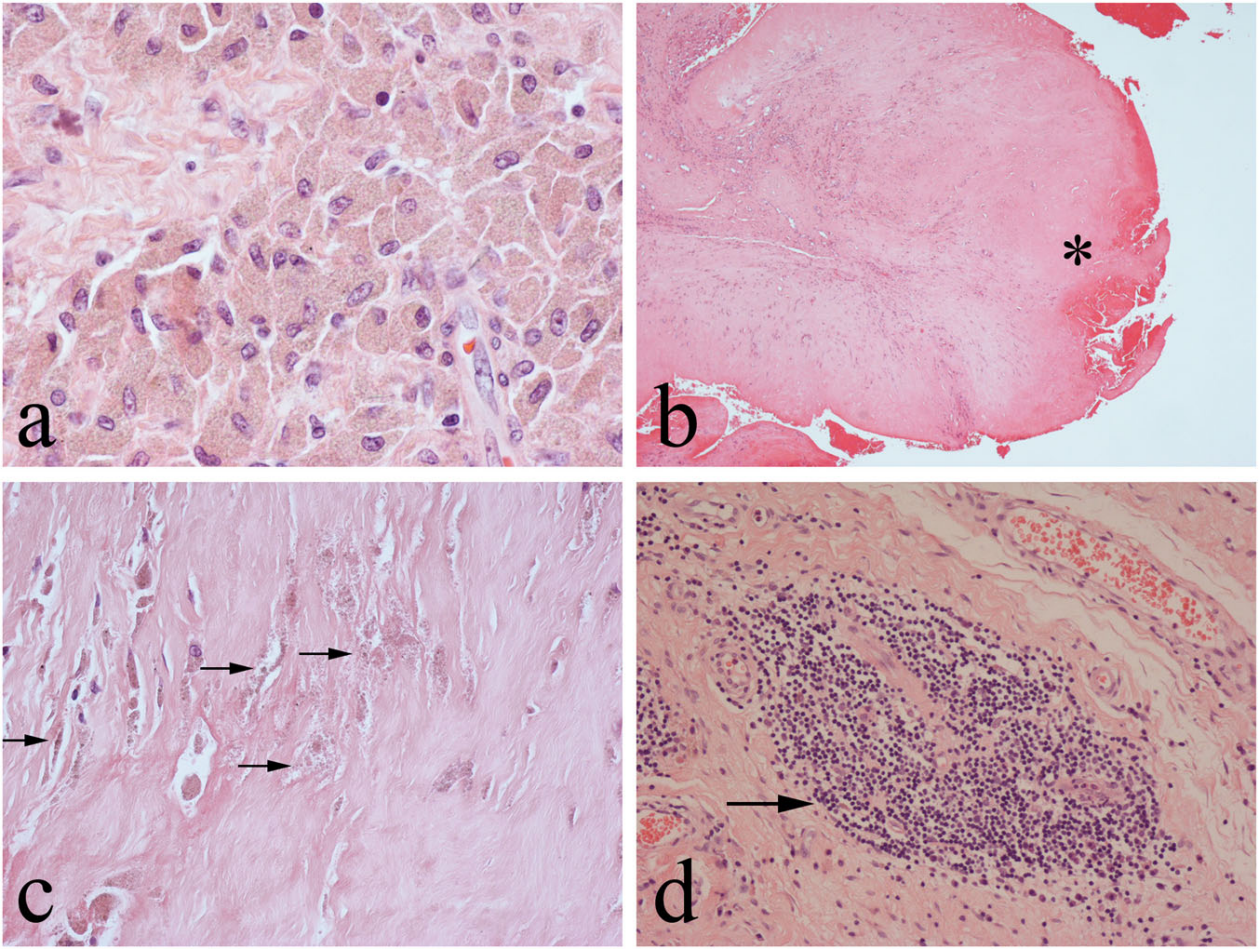
Representative HE-stained sections of MoM periprosthetic tissue showing: **a** macrophages containing fine brown corrosion Co-Cr metal wear particles; **b** necrotic superficial zone (*) on the surface of an MoM pseudotumour in which can be seen at high-power **c** viable and apoptotic/necrotic macrophages and numerous pigmented Co-Cr metal wear particles (some arrowed); **d** ALVAL with a collection of lymphoid cells (arrowed) around small vessels

Haematoxylin-eosin (HE) - stained sections of periprosthetic tissue from failed MoM hip implants showed variably necrotic and degenerate connective tissue in which there was a foreign-body macrophage response to Co-Cr particles. Most macrophages and macrophage polykaryons (giant cells) had plump cytoplasm which stained either pale pink or slate grey, but some cells had pigmented cytoplasm with fine (sand-like), yellow/brown (Co-Cr) particles (Fig. 1a). Co-Cr particles released from necrotic /apoptotic macrophages were commonly seen on the tissue surface, particularly in the necrotic superficial zone of MoM pseudotumours (Fig. 1b, c). In most cases there was also a prominent lymphoid infiltrate around blood vessels (Fig. 1d), a feature that has been termed aseptic lymphocyte-dominated vasculitis-associated lesion (ALVAL) [22].

**Fig. 2:**
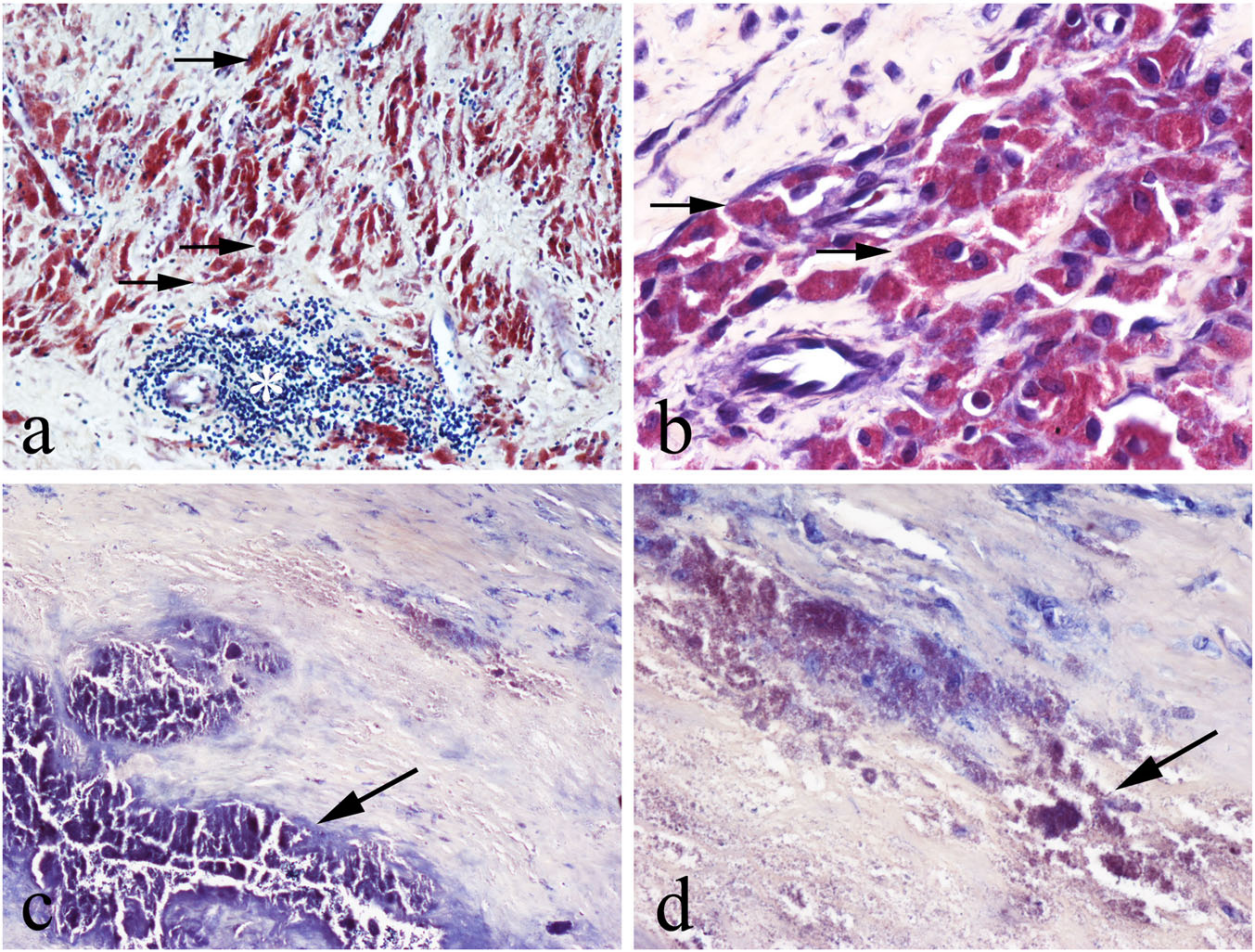
**a** Low-power and **b** High-power views of SC cytoplasmic staining of macrophages and giant cells containing metal wear (some arrowed); ALVAL lymphoid cells (asterisk) and other connective tissue elements are unstained. **c** SC staining of the superficial necrotic zone with **d** high-power view of SC-stained metal wear debris (arrowed)

**Fig. 3:**
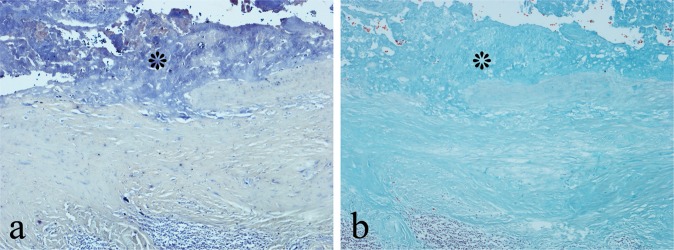
Cystic MoM pseudotumour showing: **a** blue SC staining for metal wear debris and **b** absence of (Goldners) trichrome staining for fibrin in the superfical necrotic zone (*)

In Solochrome Cyanine (SC) -stained MoM periprosthetic tissues, there was a discrete colour product (most commonly purple but ranging from magenta to blue) in the cytoplasm of macrophages and giant cells (Fig. 2a, b). On the necrotic tissue surface of pseudotumours, where apoptotic and necrotic macrophages released their cytoplasmic contents, there was a variable amount of purple staining (Fig. 2c, d); this necrotic tissue/exudate appeared to contain (Co-Cr) wear debris rather than fibrin as trichrome staining showed that fibrin was either absent or a relatively minor component in the superficial zone (Fig. 3a, b). SC stained muscle red but other cell and tissue elements, including lymphocytes and plasma cells, blood vessels, fat and collagenous fibrous tissue, were unstained. Polymethyl-methacrylate (PMMA) particles were not stained by SC.

**Fig. 4:**
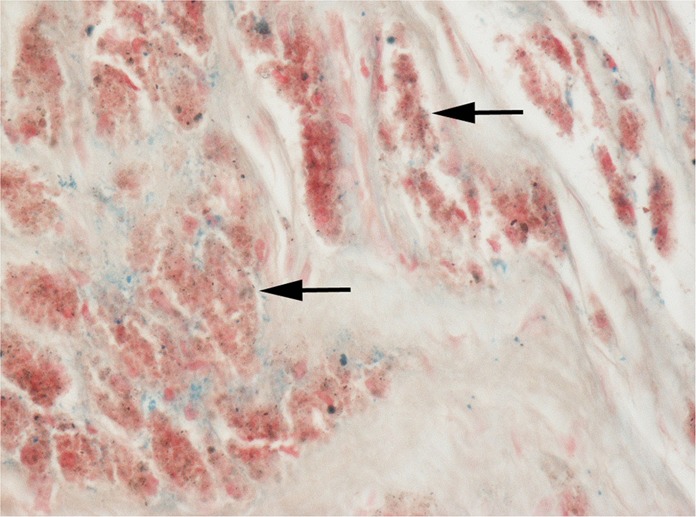
Prussian Blue staining of MoM periprosthetic tissue, showing positive (blue) staining for Fe^3+^ of some but not all pigmented foreign body macrophages and no staining of particulate Co-Cr metal wear debris (some arrowed)

Extracellular haemosiderin deposits were present in most specimens of MoM periprosthetic tissues and were highlighted by Prussian Blue (PB) staining. These deposits were SC-negative. Most macrophages were not PB-negative but cytoplasmic PB staining of some pigmented macrophages was noted; in these cells particulate Co-Cr metal wear debris was not stained by PB (Fig. 4). PB staining was not seen in the necrotic exudate covering periprosthetic tissues.

In MoM periprosthetic tissues stained with SA, macrophages and giant cells containing Co-Cr particles were unstained and there was no staining of identifiable (PB-positive) haemosiderin deposits.

### Histological staining of non-MoM periprosthetic tissue

In HE stained sections of periprosthetic tissue from failed non-MoM hip implants, there was a foreign body response to specific implant-derived wear particles. Periprosthetic tissue containing UHMWP and titanium (+/− PMMA) particles elicited a heavy macrophage and giant cell response whereas a less pronounced macrophage response to ceramic wear particles was seen.

**Fig. 5:**
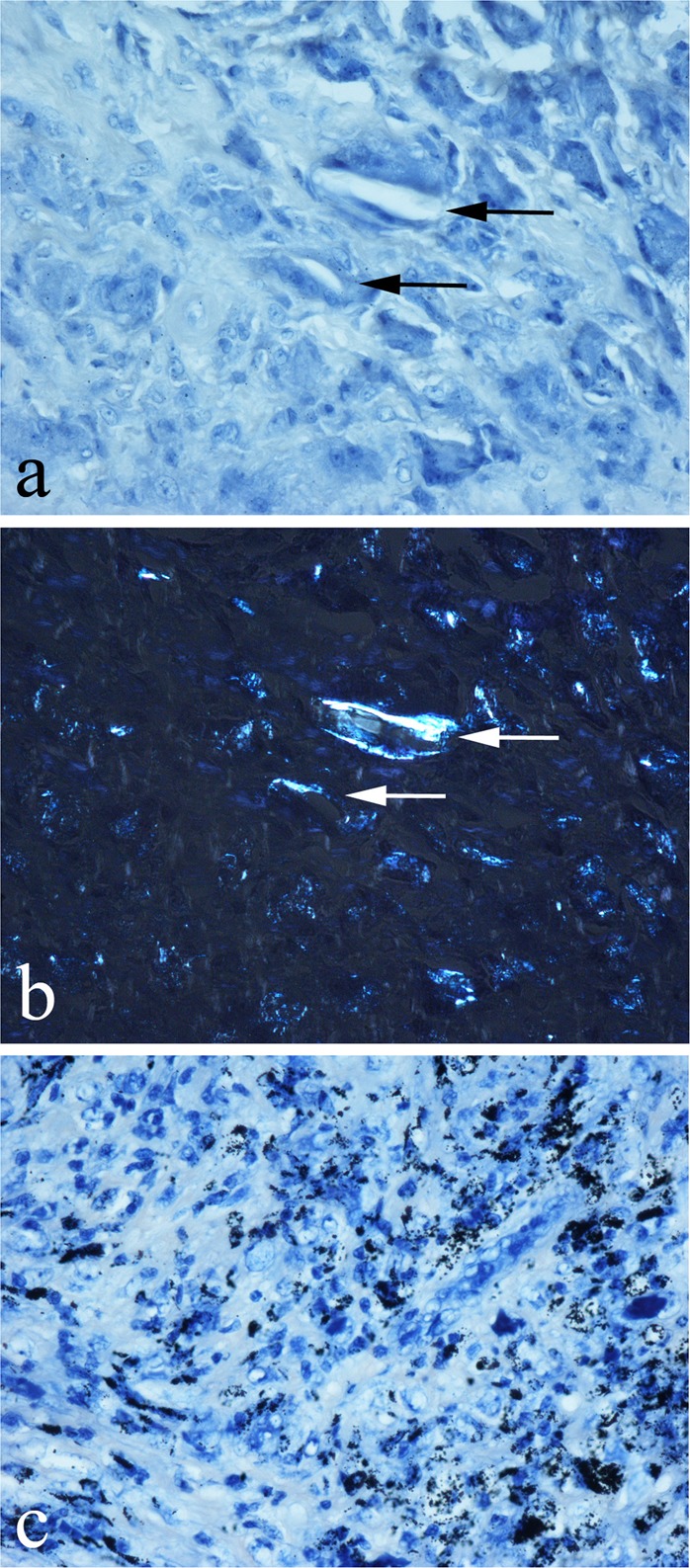
Non-MoM periprosthetic tissues from failed hip arthroplasty showing absence of SC staining of: **a** UHMWP wear particles (some arrowed) in macrophages [strongly birefringent under polarised light in **b**] and **c** black titanium particles in macrophages and giant cells

In non-MoM periprosthetic tissue, there was no SC staining of phagocytic cells that contained PMMA, UHMWP (Fig. 5a, b), titanium (Fig. 5c), or ceramic particles. Large (up to 400 µm) UHMWP particles and titanium particles were unstained by SC, the latter appearing black, as in HE-stained sections. Haemosiderin deposits in non-MoM periprosthetic tissues were stained by PB. In SA-stained non-MoM periprosthetic tissues, there was no discrete staining of macrophages or giant cells containing PMMA, UHMWP, and ceramic or titanium particles.

### Histological staining of haemosiderin in TSGCT

**Fig. 6:**
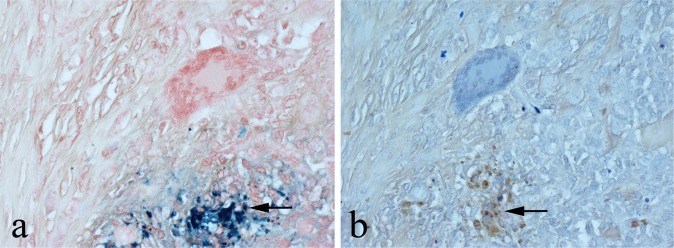
Diffuse TSGCT with Fe^3+^—containing haemosiderin deposit (arrowed), showing: **a** strong blue staining with PB but **b** no staining with SC

Haemosiderin deposition was noted on HE staining in extracellular connective tissue and phagocytic cells of the synovium in all 10 cases of diffuse TSGCT. There was strong PB staining of haemosiderin but no staining with either SC or SA, the deposits (as in HE-stained sections) appearing brown in colour (Fig. 6).

## Discussion

Metal-on-metal (MoM) hip implants are known to produce abundant Co-Cr-containing metal debris which may be in the form of wear particles, organometallic complexes, metal salts/oxides and/or free metal ions [3, 4, 11]. An adverse response in periprosthetic bone and soft tissues to metal wear debris is widely recognised as a significant cause of MoM arthroplasty failure [3, 8, 10, 23, 24]. In this study, we have shown that in paraffin-embedded histological preparations of MoM periprosthetic tissue, Solochrome Cyanine (SC) stained metal wear debris in phagocytes and in the superficial necrotic zone. SC did not stain other types of implant-derived wear particles and did not stain haemosiderin, either in periprosthetic tissues or in TSGCT.

Solochrome Cyanine (CI Mordant Blue 3; CI43820) is a polycyclic aromatic sulfonic acid which has numerous pseudonyms including eriochrome cyanine R, chromoxane cyanin R and chromoxane B [25]. SC has been employed as a differential stain to demonstrate a number of tissue elements in histology including osteoid in bone and myelin in neural tissues [17]. SC is mixed with a ferric salt for histological application and there is spectrophotometric evidence for the existence of three dye-metal complexes in the solutions of the dye containing added ferric chloride at pH 1–5 [21, 25]. The observation of blue and various shades of red in tissue stained with SC solutions containing iron (III) probably results from the formation of several red and blue complexes of the dye. The intensity of the anionic blue complex, which is a metal chelate, can be increased in any solution by raising either the pH or the iron: dye ratio. SC and related compounds have been used as reagents in the spectrophotometric identification or extraction of several trace metals, including Cr [18, 26, 27]. SC is structurally similar to CAS which has previously been reported to stain Cr in histological sections of the liver and kidney of rats injected with Cr [19]. Implant-derived soluble corrosion and particulate metal wear debris found in periprosthetic tissue contains mainly Cr^3+^ phosphate [28–30]; this metal debris is highly concentrated in the cytoplasm of macrophages as nanoparticulates [11, 31]. Macrophages and giant cells are specialised for particle phagocytosis, and it is possible that the high intracellular concentration of Cr-containing particulate wear could explain the strong cytoplasmic SC staining of these cells and the absence of staining in other cell types which are not specialised for phagocytosis. Our observation that SC strongly and selectively stains the cytoplasm of phagocytes in MoM periprosthetic tissue would be consistent with SC staining of Cr within these cells. As SC is known to react with other metals, definitive proof of this association requires confirmation by microanalytical techniques such as X-ray spectroscopy or inductively coupled plasma-mass spectrometry (ICP-MS). However, our results clearly show that SC staining is useful in the histological identification of Cr-containing wear particles as SC did not stain iron-containing haemosiderin or other metal (or polymer) wear particles derived from implant components that are commonly employed in MoM/non-MoM hip arthroplasty, including titanium, (aluminium/zirconium-containing) ceramic, UHMWPE and PMMA.

Co-Cr metal wear particles have been subcategorised into larger conventional metallic particles generated by adhesion/abrasion and smaller (mainly nano-size) corrosion metal particles which are produced by tribocorrosion, the process of degradation of the surface of metal implants due to the combined action of mechanical loading and electrochemical corrosion [3, 4, 14]. Corrosion metal particles are abundant in MoM periprosthetic tissues and have a shape which varies from round/globular to rod/needle-like ultrastructurally [2, 11, 28, 29]; these particles can appear green-yellow or yellow-brown histologically and have been shown by energy dispersive X-ray spectroscopy to have a variable metal composition with a discrete Cr-peak [13, 28, 29]. We found that SC strongly stained the cytoplasm of foreign body macrophages (and giant cells) with the resultant colour product being most commonly purple but ranging from magenta to blue. It is possible that differences in the nature of the metal wear debris in phagocytes with regard to Cr concentration, elemental/structural composition, proportion of particulate and soluble metal wear, and possibly variations in local intracellular/extracellular pH could account for differences in the SC colour product. Variable SC staining of the necrotic superficial zone was also noted and could similarly be due to differences in the nature and composition of metal wear debris in this location.

Histological identification of implant-derived wear particles is important in the diagnosis of implant failure. Individual Co-Cr particles, being nano in size, are too small to be seen under the light microscope and, even when they form visible aggregates, can be difficult to identify histologically. Our empiric observation that SC specifically stains macrophages containing metal debris in MoM periprosthetic tissue is thus likely to be of diagnostic utility in the identification of Co-Cr wear particles. It can be challenging even for experienced musculoskeletal histopathologists to identify morphologically a specific type of wear particle in periprosthetic tissue, particularly when implant components in an arthroplasty are of more than one biomaterial type; for example, in the periprosthetic tissues of MoM arthroplasties employing a titanium acetabular shell, both titanium and Co-Cr metal wear particles can be found in MoM periprosthetic tissues. We noted that titanium, as well as ceramic; PMMA and UHMWP particles were unstained by SC in non-MoM periprosthetic tissues.

Metal-on-metal implant-derived metal wear needs to be distinguished not only from other exogenous wear particles but also endogenous cell and tissue products, such as fibrin and haemosiderin. Histologically, a necrotic superficial zone on the surface of MoM periprosthetic tissues is often seen in cases of adverse reaction to metal debris (ARMD), particularly in cases of pseudotumour. Metal wear debris in this zone is derived not only from the contiguous metal implant but also from necrotic and apoptotic macrophages that have released Co-Cr wear debris as a consequence of cytotoxicity [8, 32]. We noted that there was significantly more SC-staining (for metal wear debris) than trichrome staining (for fibrin) in this superficial necrotic zone. This finding is of interest as the presence or absence of fibrin is included in some histological scoring systems of ARMD/ ALVAL [33]. Haemosiderin is commonly found in both MoM and non-MoM periprosthetic tissues where it occurs either as a consequence of previous haemorrhage from the trauma of the initial arthroplasty surgery or from implant loosening. Haemosiderin is an iron storage complex which appears in HE-stained sections as golden or honey-brown globular deposits in phagocytic cells and/or extracellular connective tissue. In HE-stained histological sections of MoM periprosthetic tissue, haemosiderin can morphologically resemble yellow/brown corrosion Co-Cr metal wear particles. SC was useful in the distinction of corrosion Co-Cr metal wear from haemosiderin as it did not stain identifiable (PB-positive) haemosiderin deposits in MoM/non-MoM periprosthetic tissue or in diffuse TSGCT, a condition characterised by significant haemosiderin deposition [34]. In the PB reaction, iron-containing potassium ferricyanide combines with ferric ions to form potassium ferric ferricyanide, an insoluble blue deposit. It has previously been noted that in MoM periprosthetic tissue haemosiderin may be present in conjunction with implant-derived wear particles in phagocytes [12, 13]. Our finding of a positive PB reaction in some phagocytic cells that contained (PB-negative SC positive) metal wear debris would be consistent with this observation. Krenn et al. [35] has postulated that homogenous weak cytoplasmic PB reactivity in MoM periprosthetic tissue macrophages could occur in response to a high metal ion load. However, Cr^3+^ is not a strong enough oxidising agent itself to oxidise the Fe^2+^ in ferrocyanide to Fe^3+^ and Yoshio et al. have shown that treatment of potassium ferrocyanide with Cr^3+^ ions produces a reddish orange colour [36].

## Conclusion

Most laboratory stains employed in histopathology are dye compounds that have been found empirically to distinguish one or more histological features of diagnostic significance. Solochrome Cyanine (SC) is likely to be useful in this regard in the histological assessment of MoM periprosthetic tissue. SC strongly and specifically stained metal wear debris in phagocytes and in the superficial necrotic zone, a feature consistent with the Cr component of metal wear being discretely stained. In addition, SC did not stain other implant-derived metal or polymer biomaterial wear particles and helpfully distinguished haemosiderin from corrosion metal wear debris.
